# Supplementary material for: Systemic Lupus Erythematosus in Hemodialysis: Survival Comparison and Mortality-Related Factors
Source: Medicina (Kaunas). 2025 Jan 17;61(1):155. doi: 10.3390/medicina61010155 (PMC11767054; doi:10.3390/medicina61010155)
Supplement: Supplementary file 1 [file medicina-61-00155-s001.zip › medicina-3405850-supplementary.pdf]

Supplementary Materials

Table S1. Cox regression analyses for survival in patients with other diseases.

Table S2. Cox regression analyses for survival in patients with DM.

Table S3. Cox regression analyses of patients with SLE based on SLE with diabetes or hypertension

Table S4. Cox regression analyses for survival after dividing patients with SLE according to the use of ISAs

Figure S1. Kaplan–Meier curves of patient survival divided by male (A) and female (B) patients.

Table S1. Cox regression analyses for survival in patients with other diseases

	Multivariate	
	**HR (95% CI)**	***P***
Age (ref: <65 years)	3.21 (3.02–3.42)	<0.001
Sex (ref: male)	0.70 (0.65-0.74)	<0.001
CCI score (per 1 score increase)	1.08 (1.07–1.09)	<0.001
Hemoglobin (per 1 g/dL increase)	0.89 (0.87–0.92)	<0.001
Serum creatinine (per 1 mg/dL increase)	0.92 (0.91–0.93)	<0.001
Systolic blood pressure (per 1 mmHg increase)	1.01 (1.00–1.01)	<0.001
Diastolic blood pressure (per 1 mmHg increase)	1.00 (0.99–1.00)	0.009
Serum calcium (per 1 mg/dL increase)	1.07 (1.04–1.10)	<0.001
Serum phosphorus (per 1 mg/dL increase)	1.03 (1.01–1.05)	0.015
Kt/V_{urea} (per 1 unit increase)	0.89 (0.79–0.99)	0.033
Serum albumin (per 1 g/dL increase)	0.49 (0.45–0.53)	<0.001
Hemodialysis vintages (per 1 day increase)	1.00 (1.00–1.00)	<0.001

The multivariate analysis was adjusted for the disease group, age, sex, CCI score, hemodialysis vintage, ultrafiltration volume, Kt/V_{urea}, hemoglobin, serum albumin, serum creatinine, serum phosphorus, serum calcium, systolic blood pressure, and diastolic blood pressure and was performed using the backward mode.

Abbreviations: HR, hazard ratio; CI, confidence interval; CCI, Charlson comorbidity index.

Table S2. Cox regression analyses for survival in patients with DM

	Multivariate	
	**HR (95% CI)**	***P***
Age (ref: <65 years)	2.16 (2.06–2.27)	<0.001
Sex (ref: male)	0.73 (0.69-0.78)	<0.001
CCI score (per 1 score increase)	1.06 (1.05–1.07)	<0.001
Hemoglobin (per 1 g/dL increase)	0.91 (0.88–0.93)	<0.001
Serum creatinine (per 1 mg/dL increase)	0.92 (0.91–0.93)	<0.001
Systolic blood pressure (per 1 mmHg increase)	1.00 (1.00–1.00)	<0.001
Serum calcium (per 1 mg/dL increase)	1.06 (1.03–1.10)	<0.001
Serum phosphorus (per 1 mg/dL increase)	1.03 (1.01–1.05)	0.011
Kt/V_{urea} (per 1 unit increase)	0.87 (0.79–0.97)	0.012
Serum albumin (per 1 g/dL increase)	0.64 (0.60–0.69)	<0.001
Hemodialysis vintages (per 1 day increase)	1.00 (1.00–1.00)	<0.001
Ultrafiltration volume (per 1 L/session increase)	1.04 (1.01–1.06)	0.009

The multivariate analysis was adjusted for the disease group, age, sex, CCI score, hemodialysis vintage, ultrafiltration volume, Kt/Vurea, hemoglobin, serum albumin, serum creatinine, serum phosphorus, serum calcium, systolic blood pressure, and diastolic blood pressure and was performed using the backward mode.

Abbreviations: HR, hazard ratio; CI, confidence interval; SLE, systemic lupus erythematosus; DM, diabetes mellitus, CCI, Charlson comorbidity index.

Table S3. Cox regression analyses of patients with SLE based on SLE with diabetes or hypertension

	Multivariate	
	**HR (95% CI)**	***P***
Ref: SLE without diabetes		
SLE with diabetes	2.00 (1.31–3.07)	0.001
Diabetes alone	1.59 (1.26–2.01)	<0.001
Ref: SLE with diabetes		
Diabetes alone	0.79 (0.55–1.14)	0.207
Ref: SLE without hypertension		
SLE with hypertension	0.98 (0.58–1.65)	0.934
Hypertension alone	1.01 (0.63–1.63)	0.953
Ref: SLE with hypertension		
Hypertension alone	1.04 (0.84–1.29)	0.741

The multivariate analysis was adjusted for the disease group, age, sex, Charlson Comorbidity Index score, hemodialysis vintage, ultrafiltration volume, Kt/V_{urea}, systolic blood pressure, diastolic blood pressure, as well as levels of hemoglobin, serum albumin, serum creatinine, serum phosphorus, and serum calcium; and it was performed using the backward mode.

Abbreviations: CI, confidence interval; HR, hazard ratio; SLE, systemic lupus erythematosus

Table S4. Cox regression analyses for survival after dividing patients with SLE according to the use of ISAs

	Univariate		Multivariate	
	**HR (95% CI)**	***P***	**HR (95% CI)**	***P***
Ref: SLE without ISAs				
SLE with ISAs	1.04 (0.80–1.36)	0.783	1.44 (0.96–2.15)	0.075
Diabetes	1.89 (1.56–2.28)	<0.001	1.65 (1.21–2.26)	0.002
Others	1.05 (0.87–1.27)	0.599	0.99 (0.73–1.35)	0.963
Ref: SLE with ISAs				
Diabetes	1.81 (1.51–2.20)	<0.001	1.15 (0.89–1.48)	0.288
Others	1.01 (0.84–1.22)	0.892	0.69 (0.53–0.89)	0.004

The multivariate analysis was adjusted for the disease group, age, sex, Charlson Comorbidity Index score, hemodialysis vintage, ultrafiltration volume, Kt/V_{urea}, systolic blood pressure, and diastolic blood pressure, as well as levels of hemoglobin, serum albumin, serum creatinine, serum phosphorus, serum calcium; and it was performed using the backward mode. Abbreviations: CI, confidence interval; HR, hazard ratio; SLE, systemic lupus erythematosus; ISA, immunosuppressive agent.

A

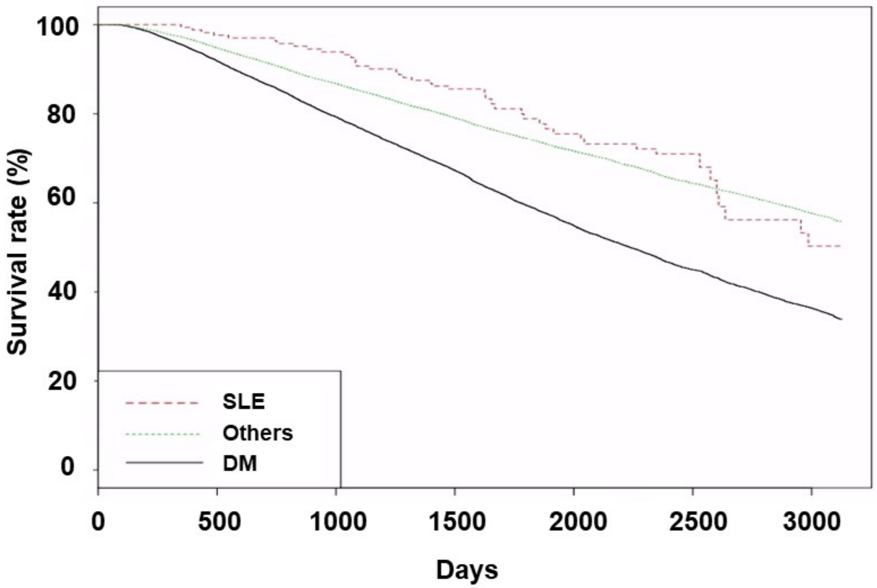

B

Figure S1. Kaplan–Meier curves of patient survival divided by male (A) and female (B) patients. For male patients, *P*-values were <0.001 for trend, <0.001 for DM vs. SLE or others, and 0.220 for SLE vs. others. For female patients, *P*-values were <0.001 for trend, <0.001 for DM vs. SLE or others, and 0.096 for SLE vs. others. Abbreviations: SLE, systemic lupus erythematosus; DM, diabetes mellitus.
